# Significance of Coronary Calcification for Prediction of Coronary Artery Disease and Cardiac Events Based on 64-Slice Coronary Computed Tomography Angiography

**DOI:** 10.1155/2013/472347

**Published:** 2013-03-17

**Authors:** Yuan-Chang Liu, Zhonghua Sun, Pei-Kwei Tsay, Tiffany Chan, I-Chang Hsieh, Chun-Chi Chen, Ming-Shien Wen, Yung-Liang Wan

**Affiliations:** ^1^Department of Medical Imaging and Intervention, Chang Gung Memorial Hospital at Linkou, Healthy Aging Research Center, College of Medicine, Chang Gung University, Taoyuan 333, Taiwan; ^2^Discipline of Medical Imaging, Department of Imaging and Applied Physics, Curtin University, Perth, WA 6845, Australia; ^3^Department of Public Health and Center of Biostatistics, College of Medicine, Chang Gung University, Taoyuan 333, Taiwan; ^4^Michael G. DeGroote School of Medicine, McMaster University, Hamilton, ON, Canada L8S 4L8; ^5^Second Section of Cardiology, Department of Internal Medicine, Chang Gung Memorial Hospital at Linkou, College of Medicine, Chang Gung University, Taoyuan 333, Taiwan

## Abstract

This work aims to validate the clinical significance of coronary artery calcium score (CACS) in predicting coronary artery disease (CAD) and cardiac events in 100 symptomatic patients (aged 37–87 years, mean 62.5, 81 males) that were followed up for a mean of 5 years. Our results showed that patients with CAD and cardiac events had significantly higher CACS than those without CAD and cardiac events, respectively. The corresponding data were 1450.42 ± 3471.24 versus 130 ± 188.29 (*P* < 0.001) for CAD, and 1558.67 ± 513.29 versus 400.46 ± 104.47 (*P* = 0.031) for cardiac events. Of 72 patients with CAD, cardiac events were found in 56 (77.7%) patients. The prevalence of cardiac events in our cohort was 13.3% for calcium score 0, 50% for score 11–100, 56% for score 101–400, 68.7% for score 401–1,000, and 75.0% for score >1000. Increased CACS (>100) was also associated with an increased frequency of multi-vessel disease. Nonetheless, 3 (20%) out of 15 patients with zero CACS had single-vessel disease. Significant correlation (*P* < 0.001) was observed between CACS and CAD on a vessel-based analysis for coronary arteries. It is concluded that CACS is significantly correlated with CAD and cardiac events.

## 1. Introduction

The pathogenesis of coronary artery disease (CAD) is a long-term atherosclerotic process that eventually leads to significant stenosis (decrease of lumen diameter by >50%) of the coronary arteries. With reports demonstrating the initial presentation of CAD being acute myocardial infarction or sudden cardiac death in 50% of patients [1], increasing efforts have been made to establish risk factors that can assess patient risk for future coronary events. Unfortunately, the success of conventional risk factors, such as the Framingham Risk Score, clinical examination, and stress testing, have been limited in their ability to predict the occurrence of CAD, especially among patients within the intermediate risk group [2].

Coronary artery calcium score (CACS) has been regarded as a potential tool to improve risk stratification and predict cardiac events. It has been recognized as a surrogate marker for atherosclerotic plaque burden and holds the advantages of directly visualizing and precisely locating the plaques using computed tomography (CT) [3, 4]. Using Agatston calcium scoring [5], CACS can also be quantified, allowing for a direct individual assessment of each patient, unlike conventional risk factors that only provide a statistical probability for patients developing CAD. A growing number of reports have emerged supporting the vital use of CACS in the assessment of cardiac event risk stratification [3, 6]. Conventional coronary angiography (CCA) is the gold standard in diagnosing CAD due to its superior spatial and temporal resolution, thus enabling accurate assessment of the degree of coronary stenosis. However, this procedure remains invasive, expensive, and inconvenient for patients. CACS, on the other hand, is most commonly quantified using CT, which is widely used in routine clinical practice as a noninvasive technique.

The vast majority of studies describing the prognostic value of coronary calcification were mainly done in the Western countries [7–10]. Related studies reported from Asian country are relatively scarce [11, 12]. The healthcare system, populations, and disease patterns in Asia differ from Western countries [13]. Prevalence of coronary calcification is different in Caucasian, Chinese, Hispanic, and African populations by figures of 70.4%, 59.6%, 56.5%, and 52.1%, respectively. Compared with Caucasians, the relative risk of death was 2.97 in Africans, 1.58 in Hispanics, and 0.85 in Chinese [2]. In this report from an Asian country, we aim to validate the relationship between CACS, CAD, and cardiac events by using 64-multislice computed tomography (64-MSCT) with CCA as the gold standard.

## 2. Materials and Methods

### 2.1. Patients

Medical records of CCA and CACS over 2 years (2006–2008) from Chang Gung Memorial Hospital in Taiwan were retrospectively reviewed of 100 symptomatic patients suggestive of CAD. These symptomatic patients included 81 men, with ages ranging from 37 to 87 (mean 62.5) years. The main symptoms prior to CCA and 64-MSCT testing included chest tightness (*n* = 57), chest pain (*n* = 44), radiating pain (*n* = 26), dyspnea (*n* = 38), and cold sweats (*n* = 25). Risk factors for CAD that were apparent among the patient population included hypertension (*n* = 61), hypercholesterolemia (*n* = 27), hypertriglyceridemia (*n* = 36), smoking history (*n* = 14), diabetes mellitus (*n* = 22), and obesity or overweight (*n* = 33). All patients underwent CCA and MSCT for CACS. The interval between the testing of CCA and 64-MSCT ranged from 0 to 89 (mean 9.16 ± 16.82) days, where the interval was less than two weeks in 79% of all cases. For assessing cardiac events after cardiac CT, 98 patients could be followed up for a mean of 5 years (range 46.7–72.9 months). Each subject was recorded to have at least one cardiac event, by definition as occurrence of either unstable angina requiring revascularization or cardiac death (caused by acute myocardial infarction, ventricular arrhythmias, or refractory heart failure). The institute review board approved the study, and written informed consent was obtained from all patients undergoing CCA and CT. Patients were excluded if they had contrast medium allergy, impaired renal function, history of coronary bypass surgery, and arrhythmia.

### 2.2. Conventional Coronary Angiography (CCA)

CCA was referred to all patients with suspected CAD, as described by their symptoms, risk factors, and experiencing at least one cardiac event. CCA was done accordingly to the standard Seldinger's technique on an angiographic machine (Integris BH3000, Philips, Eindhoven, The Netherlands) by femoral approach. Cardiologists who had no prior knowledge of MSCT findings quantitatively analyzed the severity of coronary stenosis. The minimal lumen diameter was measured in projections showing the most severe narrowing. The degree of stenosis was classified into four categories: (1) no stenosis, (2) minimal or mild stenosis (≤50%), (3) moderate stenosis (50%–70%), and (4) severe stenosis (>70%). CAD was defined when lumen diameter reduction was greater than 50% (moderate or severe stenosis).

### 2.3. Cardiac CT Imaging Protocol

All CT scans were performed on a 64-slice scanner with a 0.4 s rotation time (Aquilion Multi-64-slice system, Toshiba Medical Systems). Nonenhanced CT scan for calcium scoring was performed from the level of tracheal bifurcation to the diaphragm using the following parameters: 120 KVp, 300 mA, 0.25 s, slice thickness of 3 mm, and intervals of 3 mm. The calcium scores of each area at each vessel were calculated at an offline commercially available workstation with dedicated software (Software Vitrea 2 V3.9.0.1, MN, USA) and the scores were quantified by the scoring algorithm proposed by Agatston et al. [5], and calcium scores were divided into the following categories: 0, 1–10, 11–100, 101–400, 401–1000, and ≥1000.

### 2.4. Statistical Analysis

Continuous variables were expressed as mean and standard deviations. For both patient-based and vessel-based analyses, Kruskal-Wallis test was used to analyze whether the CACS were related to the degree of coronary artery stenosis. Mann-Whitney *U* test was used to investigate the correlation between the CACS and the presence of CAD. Two-sample independent *t*-test was used to analyze the correlation between CACS and cardiac events. Chi-square test was used to assess the correlation between the cardiac events and categorical variables (age, gender, risk factors including hypertension which was defined as blood pressure > 130/90 mm Hg, diabetes mellitus, smoking, body mass index, and hypercholesterolemia). Event-free survival curves were constructed using the Kaplan-Meier method to account for censored survival times and compared with the log-rank test. A *P* value of less than 0.05 was considered statistically significant.

## 3. Results

### 3.1. Significant Correlation between CACS and CAD on a Patient Basis

Of 100 symptomatic patients, CCA revealed CAD (stenosis >50% in diameter) in 72 patients, while the remaining 28 patients had no CAD (stenosis ≦ 50%) (Table 1). Among the patients diagnosed with CAD, 57 had severe stenosis and 15 had moderate stenosis. On the other hand, 9 patients had minimal or mild stenosis and 19 patients had no stenosis. Our findings indicated that: (1) there was a significant increase in mean calcification with increasing severity in stenosis, (2) the variability of calcium scoring was high within each group, and (3) the overall calcium score in patients with CAD was significantly higher than those without CAD (1450.42 ± 3471.24 and 130 ± 188.29, resp.; *P* < 0.001) (Table 1). This suggests that patients with extensive coronary calcification have a higher probability of moderate stenosis and, thus, are more likely to have CAD. Patients with a calcium score of 0, 11–100, 101–400, 401–1000, and >1000 had a 20%, 62.5%, 76%, 75%, and 100% prevalence of CAD, respectively (Table 1). A significant correlation was confirmed between the degree of stenosis and calcium score (*P* < 0.001) (Table 1).

### 3.2. Significant Correlation between CACS and CAD on a Vessel Basis

**Figure 1:**
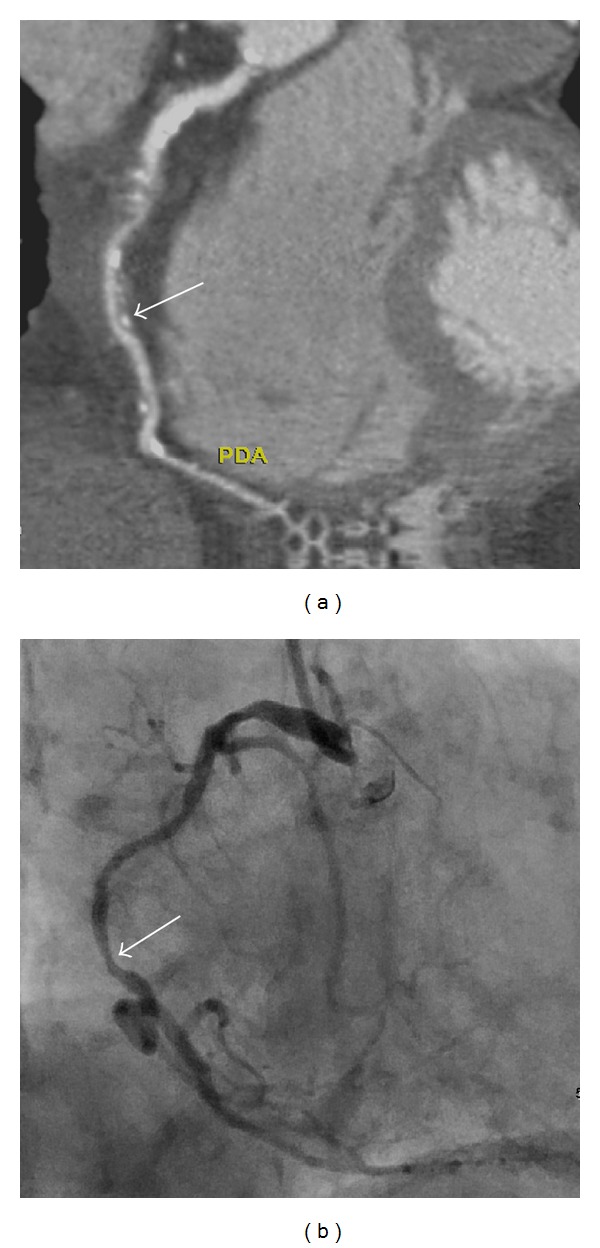
A 86-year-old female with two-vessel coronary artery disease, total calcium score was 1278. The calcium score was 325 over the right coronary artery (RCA). (a) A computed tomographic angiogram shows mixed plaques over the middle third of RCA with 54% stenosis (white arrow). (b) Conventional coronary arteriogram confirms the moderate stenosis over the proximal as well as middle (arrow) third of RCA.

Among the 100 patients, a total of 400 vessels were analyzed in which CAD (severe or moderate stenosis) was found in 131 vessels, and no CAD was found in the remaining 269 vessels (Table 2). Of the 131 vessels with significant stenosis, 38 were in the right coronary artery (RCA) (29%), 13 were in the left main artery (LM) (9.9%), 49 were in the left anterior descending (LAD) (37.4%), and 31 were in the left circumflex artery (LCX) (23.7%). As expected, CACS was significantly greater in patients with CAD than those without CAD, with the corresponding CACS being 1017.63 ± 3039.32 and 134.18 ± 297.28, respectively, in the RCA (*P* < 0.001), 456.76 ± 515.48 and 176.71 ± 365.09, respectively in the LAD (*P* < 0.001), and 381.74 ± 887.48 and 86.41 ± 205.94, respectively, in the LCX (*P* < 0.001). CACS was lower in the LM compared to all other blood vessel in both CAD and non-CAD patient groups. Also, marginal significant findings between the CACS and patients with and without CAD were noticed in the LM (159.31 ± 206.48 and 58.25 ± 124.86, resp.; *P* = 0.055). Our results revealed a positive correlation between greater calcium score and the frequency of multivessel disease (Table 1). Specifically, all patients with multivessel disease (CAD in two or three arteries) had a calcium score that was at least greater than 100 and patients with CACS > 1000 had a 100% incidence of CAD (*P* < 0.001) (Figure 1).

The sensitivity, specificity, positive predictive value (PPV), negative predictive value (NPV), and accuracy of CACS at different score levels are analyzed in Table 3. Using CCA as the gold standard, in patient-based analysis, CACS of 11–100 yielded the highest sensitivity (95.8%), NPV (80%), and accuracy (80%). CACS of over 1000 revealed the greatest specificity (100%) and PPV (100%). For each coronary artery, CACS of 1–10 yielded the highest sensitivity (91.6%) and NPV (91.9%), and CACS of >1000 revealed the highest specificity (98.9%) and PPV (83.3%). The greatest accuracy (72.8%) was obtained with CACS of 401–1000. 

### 3.3. Zero CACS Scoring Cannot Exclude the Presence of CAD

**Figure 2:**
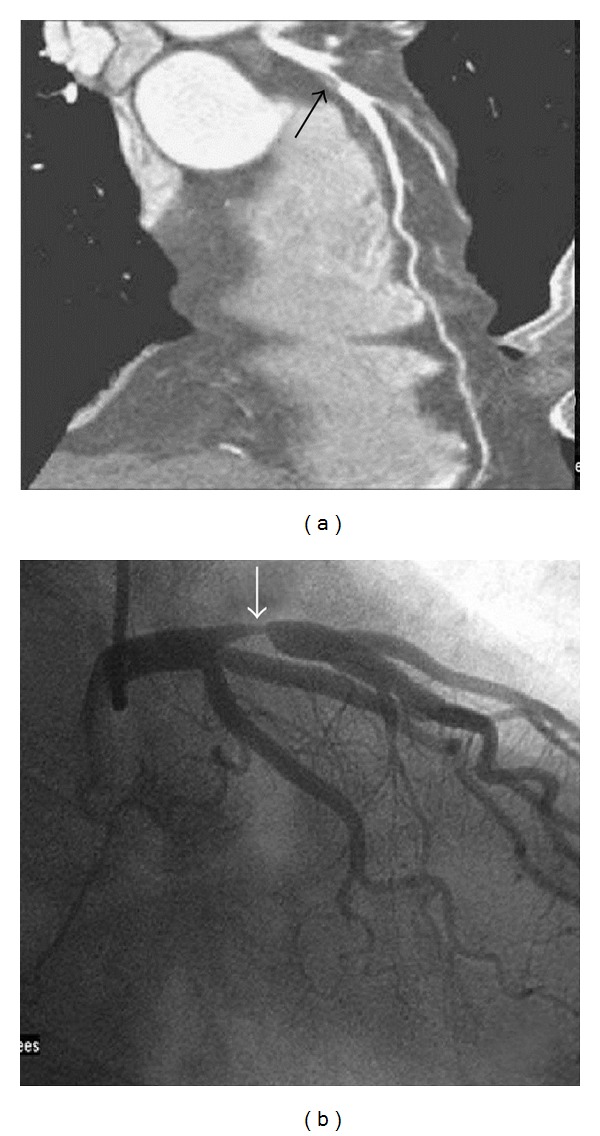
A 53-year-old male with zero calcium score. (a) A computed tomographic angiogram shows a soft plaque at the left anterior descending artery (LAD) (black arrow) with severe stenosis. (b) Conventional coronary arteriogram confirms the severe stenosis over the proximal third of the LAD (white arrow).

**Figure 3:**
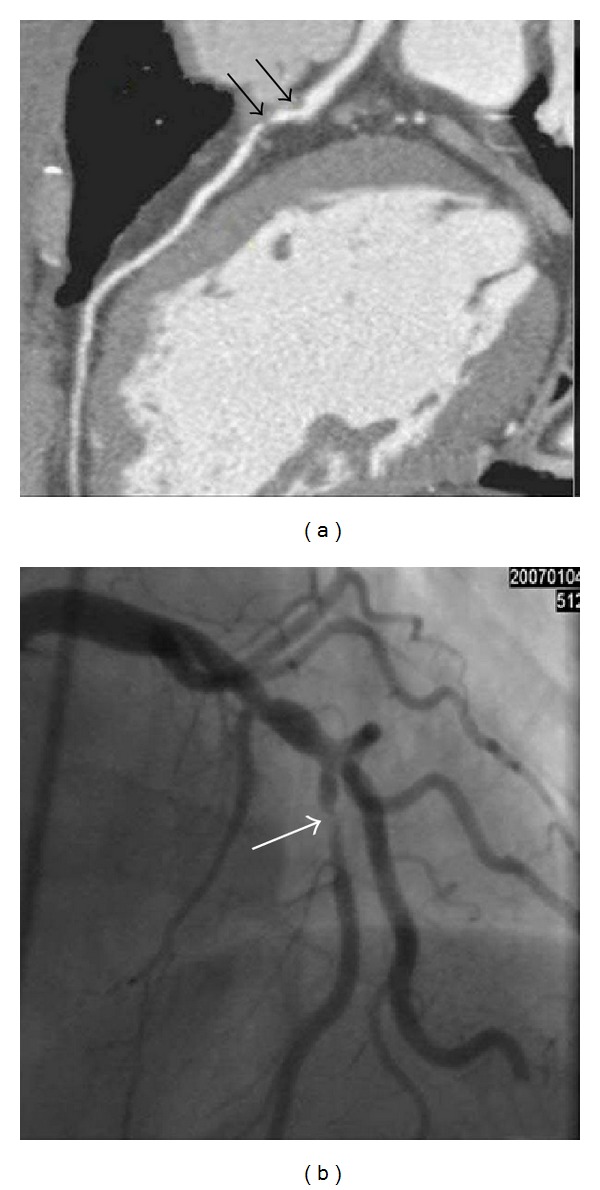
A 66-year-old male with zero calcium score. (a) A computed tomographic angiogram shows soft plaques (black arrows) at the left anterior descending artery (LAD) with severe stenosis. (b) Conventional coronary arteriogram confirms the severe stenosis of the LAD (white arrow).

A total of 15 patients did not have coronary calcification, with 3 (20%) of them having CAD (Table 1), indicating that the complete absence of coronary calcium did not exclude the presence of CAD. Following the analysis of the 3 patients with zero CACS, all were found to have single-vessel CAD primarily involving the LAD (Table 1). All three patients were confirmed to have soft plaques on CT angiograms (Figures 2(a) and 3(a)). One patient had moderate stenosis and 2 had severe stenosis confirmed by CCA (Figures 2(b) and 3(b)).

### 3.4. A Significant Correlation between CACS and Cardiac Events

Of 98 patients with a mean followup of 5 years, cardiac events occurred in 56 (57.1%) patients which were all associated with CAD. These cardiac events included two cardiac deaths (no revascularization) and 54 revascularization (Table 1) including 3 subsequent cardiac deaths. Of 72 patients with CAD, cardiac events were encountered in 56 (77.7%) subjects. Patients with cardiac events had statistically significant higher CACS than those without cardiac events: 1558.67 ± 513.29 versus 400.46 ± 104.47 (*P* = 0.031). Cardiac events were not significantly related to patient age (*P* = 0.576), gender (*P* = 0.775), hypertension (*P* = 0.800), body mass index (*P* = 0.815), smoking (*P* = 1.000), and hypercholesterolemia (*P* = 0.410) but closely related to diabetes mellitus (*P* = 0.021).

**Figure 4:**
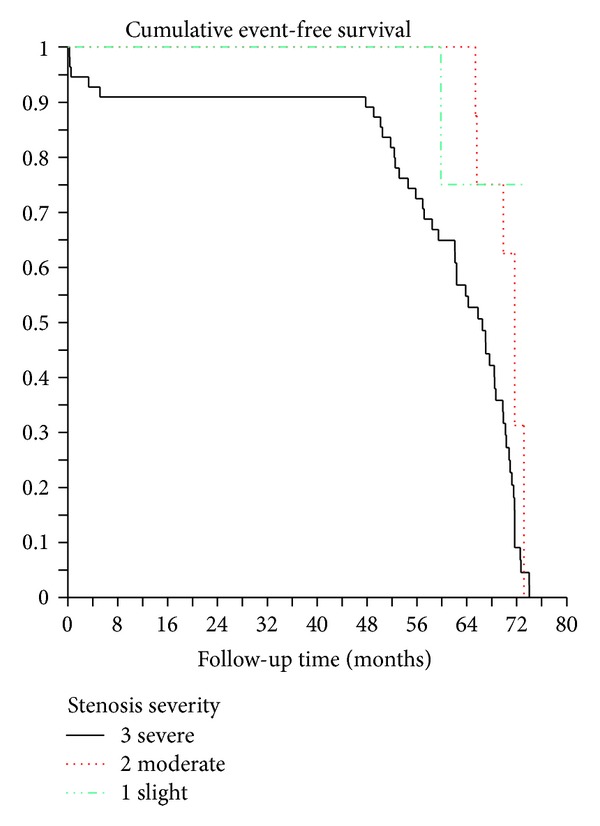
Cumulative event-free survival curves by Kaplan-Meier analysis according to the degree of coronary stenosis.

**Figure 5:**
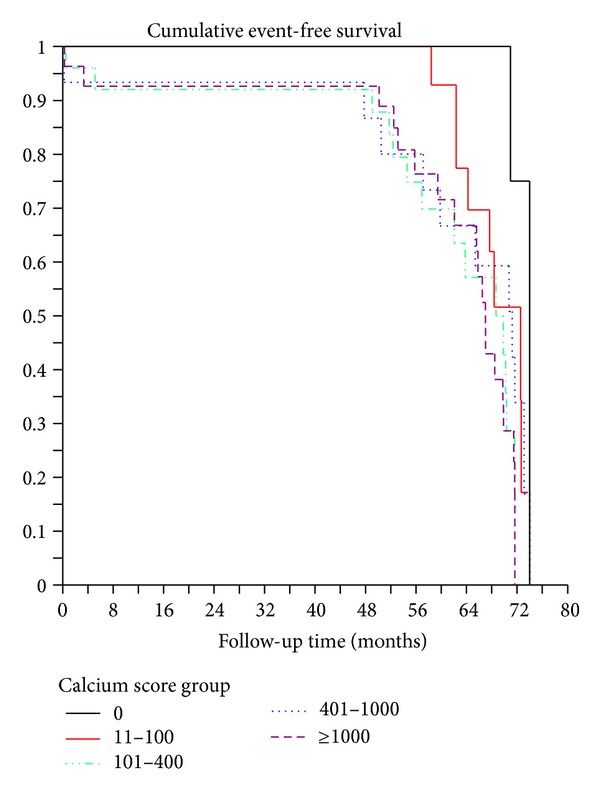
Cumulative event-free survival curves by Kaplan-Meier analysis according to the categories of coronary artery calcium score.

Figure 4 shows significant association of coronary stenosis with major adverse cardiac events. The cumulative event-free subjects curves according to calcium score categories are reported in Figure 5. As shown in these two figures, significant associations were found between the degree of coronary stenosis and calcium scores and the occurrence of cardiac events.

## 4. Discussion

The strength of our study is that it provides prognostic information of CACS for cardiac events based on a mean followup of 5 years. We also identify the clinical value of using CACS for determining the presence and degree of CAD, although a zero CACS cannot exclude the presence of CAD.

Up to 50% of CAD patients initially suffer from acute myocardial infarction (AMI) or sudden death [1], and the severity of these hard cardiac events has prompted a greater emphasis on preventative care. Thus, scoring tools that consider demographic and clinical characteristics are used to stratify patients into low-, intermediate-, and high-risk for developing CAD. In addition to the Framingham Risk Score (FRS) that uses a multivariable statistical model to predict a patient's 10-year risk for future cardiovascular events, other tools include clinical examinations, stress testing, C-reactive protein, and family history of CAD. Nonetheless, such prediction models for CAD have limitations [13]. Akosah et al. [14] conducted a survey consisting of a group of 222 asymptomatic patients who suffered from their first AMI and found that 75% of them would not have been considered for therapy according to conventional risk factors. Other studies have shown that testing can only predict 60–65% of cardiovascular events, leaving up to one-third of patients suffering from a hard cardiac event in the absence of these risk factors [15]. Such shortcomings lie in that conventional risk factors only provide a statistical probability of patients developing CAD, rather than a direct individual assessment [16]. Patients in the intermediate risk group are especially affected, as they are left untreated due to cost inefficiency and their asymptomatic condition results in poor compliance to lifestyle change [2].

The prognostic value of CACS over clinical and laboratory data has been previously studied in a large cohort of patients [12, 16, 17]. These studies showed that an excellent survival was achieved in patients with a zero CACS, but increased cardiac events were closely associated with higher CACS (<400). This is confirmed in our study as we found the similar probability of 5-year cardiac events, which was 75% for CACS > 1000 and 13.3% for CACS = 0. The occurrence of cardiac events for the patients with a zero CACS is significantly higher than that reported by Hou and others [12, 16, 17]. This could be caused by the small sample size in our study. We also found the correlation of severity of CAD with adverse cardiac events, with severe coronary stenosis leading to 86% cardiac events, and only 11.1% for patients with mild degree of coronary stenosis. This indicates incremental prognostic value of adding coronary stenosis to CACS over clinical risk factors.

A growing number of reports have emerged supporting the use of CACS as a diagnostic tool for asymptomatic patients at intermediate risk for CAD and the diagnosis of CAD in symptomatic patients [18, 19]. A study by Raggi et al. [19] concluded that there was a greater incidence of hard cardiac events (AMI and sudden death) in asymptomatic patients who had calcium scores greater than the 75th percentile when compared with their age- and sex-matched controls. Another study reported the odds ratio of hard cardiac events in asymptomatic patients with Agatston CACS scores <100, 100–400, and >400 to be 2.1, 4.2, and 7.2, respectively [20]. Among symptomatic patients, Georgiou et al. [7] reported that calcium score values were significantly related to occurrence of hard cardiac events (*P* < 0.001) and all cardiovascular events (*P* < 0.001), whereby patients with CACS in the upper third and fourth quartiles (greater than the 75th percentile) were 13.2 times more likely to suffer from an event than those with zero or low scores (0 to 25th percentile). Furthermore, Detrano et al. [9] have reported that coronary calcium score is a strong predictor of incident coronary heart disease events (MI, death due to CAD) among four racial groups (Caucasian, African, Hispanic, and Chinese) in the United States. In that study, the risk of coronary events associated with increasing CACS had a hazard ratio (95% CI) of 1.00 for nondetectable calcium. For CACS of 1–100, 101–300, >300, the hazard ratio was 3.89 (1.71–8.79), 7.08 (3.05–16.47), and 6.84 (2.93–15.99), respectively. Chinese people had a hazard ratio (95% CI) for the risk of coronary heart disease with CACS of 1.25 (*P* = 0.11) compared to the Caucasian people who had a hazard ratio of 1.17 (*P* < 0.005). Our findings are in line with these studies confirming the prognostic value of CACS in a group of symptomatic patients.

According to a report by Budoff et al. [21], when compared with individuals without calcium as a hazard ratio of 1, a calcium score between 1 and 100 was associated with a “hazard ratio” for major coronary events of 3.9, a score between 101 and 300 with a “hazard ratio” of 7.1, and a score of more than 300 with a “hazard ratio” of 6.8. In this study, the prevalence of cardiac events was 13.3% for calcium score 0, 50% for score 11–100, 56% for score 101–400, 68.7% for score 401–1,000, and 75% for score >1000. The mean of CACS in our cohort with cardiac events (1559) was 3.9 times higher than that of cohort without cardiac event (400). In addition to higher CACS, the study also revealed significant correlation (*P* = 0.021) between the diabetes mellitus and cardiac events. It has been reported that type 2 diabetics with a CACS > 100 are expected to have an increased frequency of ischemia in myocardial perfusion imaging; the risk of all-cause mortality was higher in diabetics than in nondiabetics for any degree of CS [22]. All our patients with a CACS above 1000 had CAD, calcium score higher than 1000 is associated with increased specificity (100%) but decreased sensitivity (39.4%). Larger angiographic studies using electron beam tomography and electron beam computed tomography have reported similar findings [22, 23].

A previous study by Budoff et al. [24] investigated the distribution of calcification within the major coronary arteries to determine the severity and extent of angiographic disease. In another algorithmic model, Schmermund et al. [25] utilized calcium scoring to distinguish patients with or without 3-vessel and/or left main CAD. While recent studies have found a moderate correlation between CACS and the incidence of atherosclerotic disease on vessel-based analysis (*r* = 0.521) [11], our study reveals more comprehensive findings. We demonstrate (1) a statistically significant correlation between the degree of stenosis and calcium score in the RCA, LAD, and LCX (*P* < 0.001) and (2) a significantly higher CACS in patients with CAD than those without CAD in the three aforementioned coronary arteries (*P* < 0.001). Only the LM revealed nonsignificant correlation with respect to CACS and the presence of CAD, although the relationship between stenosis and calcium scoring was close to significance (*P* = 0.055). This finding may be a result of the left main artery bifurcating into the LAD and LCX, and any calcification near this junction could be assigned to varying branches. Such difficulty in assigning calcifications to a single artery could obscure the reported CACS in different blood vessels.

Although the presence of coronary artery calcium is associated with a greater risk of cardiovascular events, its ability to predict future coronary events is not absolute. A zero calcium score only reflects the absence of atherosclerotic lesions with calcified plaques greater than 1 mm in diameter, leaving noncalcified and lipid-laden “vulnerable” plaques to be present in the absence of CACS [26]. Furthermore, any identified calcification only reflects approximately 20% of the total atherosclerotic plaque burden, overlooking any soft plaques that may cause CAD [27]. Nonetheless, the absence of CAC is associated with a very low probability of significant stenosis and future cardiovascular events.

A systematic review of 49 studies revealed that the frequency of cardiovascular event among patients with zero CACS was 0.56% in asymptomatic and 1.8% in symptomatic patients [28]. This review also found CACS to have a negative predictive value as high as 99% for ruling out acute coronary syndrome [29]. Similarly, another series reported that obstructive CAD was found in 7% of patients with zero CACS and in 17% of patients with low CACS (1–100) [30].

In our study, on a per-patient basis, 20% of patients (3 out of 15) with zero CACS had single-vessel CAD. Further analysis revealed that these patients had soft plaques, which was the cause of severe stenosis at the proximal LAD. This percentage is greater than what has been previously reported because the present study population was limited in size and focused on symptomatic patients, resulting in a greater pretest probability. To address the conflict of the prognostic value of a zero calcium score, future studies investigating patient populations of varying pretest probability for CAD and clinically relevant end points (rather than an angiographic end point) are needed. Thus, despite CACS' predictive power, the occurrence of cardiac events in patients with negative calcium scores suggests that CACS should not be used as a single-decision diagnostic parameter for CAD.

There are several limitations in our study. First, CACS cannot be used to assess noncalcified soft plaques or calcified plaques that are less than 130 HU in density. Noncalcified plaques with density less than 30 HU and positive remodeling are significant predictors of acute coronary syndrome [31]. Second, the patient number of 100 is relatively small in our cohort; however, this was compensated by statistical analysis. Our patients with CAD and cardiac events had significantly higher calcium score than those without CAD (*P* < 0.001) and cardiac events (*P* = 0.031), respectively.

Third, our study included symptomatic patients who underwent clinically relevant 64-MSCT and subsequent CCA. We acknowledge the subsequent selection and verification biases that could have led to the positive correlation between CACS and angiography findings. Ideally, this bias could have been avoided by randomly assigning patients that had undergone 64-MSCT CACS for verification of CAD through conventional angiography, regardless of clinical signs or symptoms. However, it would be unethical to ask asymptomatic patients to undergo unnecessary CCA due to its invasive nature. Furthermore, since our study only focuses on symptomatic patients, our data can only suggest a similar relationship between CACS and CAD to exist in asymptomatic patients. The study also does not assess if any subjects belonged to the intermediate risk group. Future studies would benefit from investigating the correlation among CACS, CAD, and clinical or Framingham's risks factors in each patient.

In conclusion, this study further confirms the significant relationship between the CACS and the prevalence of cardiac events and the presence of CAD on a vessel-based in addition to a patient-basis analysis. The prevalence of cardiac events was significantly increased with an increase of CACS. Increased CACS (>100) was also associated with an increased frequency of multivessel disease and patients with CACS > 1000 had a 100% incidence of CAD. Although our data supports calcium screening as an additional filter before coronary angiography in symptomatic patients, a zero CACS could not exclude the presence of significant CAD.

## Figures and Tables

**Table 1:** The correlation between calcium scoring and degree of stenosis, coronary artery disease (CAD) and cardiac events.

Calcium scoring	0	1–10	11–100	101–400	401–1000	≧1001	
Number of cases	15	0	16	25	16	28	
Cardiac events^+^ (*n* = 56)	2 (13.3%)	0	8 (50%)	14 (56%)	11 (68.7%)	21 (75%)	
	Degree of stenosis (*n* = 100)						Mean ± SD

0 = no (*n* = 19)	10 (66.7%)	0	4 (25%)	3 (16%)	2 (12.5%)	0	87.32 ± 156.52*
1 = minimal or mild (*n* = 9)	2 (13.3%)	0	2 (12.5%)	3 (12%)	2 (12.5%)	0	220.11 ± 225.99*
2 = moderate (*n* = 15)	1 (6.7%)	0	1 (6.3%)	4 (12%)	3 (18.8%)	6 (21.4%)	1143.87 ± 1284.63*
3 = severe (*n* = 57)	2 (13.3%)	0	9 (56.2%)	15 (60%)	9 (56.2%)	22 (78.6%)	1531.09 ± 3851.32*

No CAD = 0 + 1 (*n* = 28)	12	0	6	6	4	0	130 ± 188.29*
CAD = 2 + 3 (*n* = 72)	3	0	10	19	12	28	1450.417 ± 3471.24*

	Coronary artery disease (*n* = 72)						

One vessel (*n* = 33)	3 (100%)	0	10 (100%)	8 (42.1%)	6 (50%)	6 (21.4%)	
Two vessels (*n* = 26)	0	0	0	8 (42.1%)	3 (25%)	15 (53.6%)	
Three vessels (*n* = 13)	0	0	0	3 (15.8%)	3 (25%)	7 (25%)	

*Statistically significant (*P* < 0.001).

^+^Followup for cardiac events was successful in 98 of 100 patients.

**Table 2:** Calcium score in vessel-based distribution of coronary artery stenosis or coronary artery disease (CAD).

Degree of stenosis	RCA (*n* = 100)		LM (*n* = 100)		LAD (*n* = 100)		LCX (*n* = 100)	
	*n*	Mean ± SD	*n*	Mean ± SD	*n*	Mean ± SD	*n*	Mean ± SD
0 = no	51	116.53 ± 303.26*	81	52.38 ± 121.98	37	162.57 ± 394.78*	59	78.47 ± 196.89*
1 = minimal or mild	11	216 ± 242.06*	6	137.50 ± 135.44	14	214.07 ± 270.84*	10	133.20 ± 246.96*
2 = moderate	13	289 ± 405.06*	6	135.71 ± 217.82	15	673.67 ± 270.84*	8	113.38 ± 104.17*
3 = severe	25	1344.52 ± 3637.06*	7	186.83 ± 188.72	34	361.06 ± 415.86*	23	475.09 ± 1011.95*

No CAD = 269	62	134.18 ± 297.28*	87	58.25 ± 124.86	51	176.71 ± 365.0*	69	86.41 ± 205.9*
CAD = 131	38	1017.63 ± 3039.3*	13	159.31 ± 206.48	49	456.76 ± 515.4*	31	381.74 ± 887.4*

RCA: right coronary artery, LM: left main coronary artery, LAD: left anterior descending artery, and LCX: left circumflex artery.

*Statistically significant (*P* < 0.001).

**Table 3:** Sensitivity, specificity, positive predictive value (PPV), negative predictive value (NPV), and accuracy of calcium scoring in assessing coronary artery disease under patient-based (PB) and vessel-based (VB) analyses.

Calcium scoring	0		1–10		11–100		101–400		401–1000		≧1001	
	PB	VB	PB	VB	PB	VB	PB	VB	PB	VB	PB	VB
Sensitivity	100%	100%	Nil	91.6%	95.8%	86.3%	81.7%	61.1%	56.3%	33.6%	39.4%	11.5%
Specificity	0%	0%	Nil	46.1%	41.4%	53.2%	62.1%	77.3%	86.2%	91.8%	100%	98.9%
PPV	32.8%	71%	Nil	45.3%	80%	47.3%	84.1%	56.7%	90.9%	66.7%	100%	83.3%
NPV	Nil	Nil	Nil	91.9%	80%	88.8%	58.1%	80.3%	44.6%	74%	40.3%	69.6%
Accuracy	32.8%	71%	Nil	61%	80%	64%	76%	72%	65%	72.8%	57%	70.3%

Nil: no patients in respective group.
